# An algorithm recommendation for the pharmacological management of allergic rhinitis in the UK: a consensus statement from an expert panel

**DOI:** 10.1038/s41533-016-0001-y

**Published:** 2017-01-23

**Authors:** Brian Lipworth, Jon Newton, Bhaskar Ram, Iain Small, Jürgen Schwarze

**Affiliations:** 1 Ninewells Hospital and Medical School, Scottish Centre for Respiratory Research, Dundee, Scotland UK; 2 Forth Valley Royal Hospital, Forth Valley Health Board, Larbert, Scotland UK; 3 Aberdeen Royal Infirmary, Grampian Health Board, Aberdeen, Scotland UK; 4 Peterhead Surgery, Grampian Health Board, Peterhead, Scotland UK; 5 Child Life and Health, The University of Edinburgh, Edinburgh, Scotland UK

## Abstract

Allergic rhinitis is a frequent presenting problem in primary care in the UK, and has increased in prevalence over the last 30 years. When symptomatic, patients report significant reduction in their quality of life and impairment in school and work performance. Achieving adequate symptom control is pivotal to successful allergic rhinitis management, and relies mostly on pharmacotherapy. While it is recognised that most mild-moderate allergic rhinitis symptoms can be managed successfully in primary care, important gaps in general practitioner training in relation to allergic rhinitis have been identified. With the availability of new effective combination therapies, such as the novel intranasal formulation of azelastine hydrochloride and fluticasone propionate in a single device (Dymista®; Meda), the majority of allergic rhinitis symptoms can be treated in the primary care setting. The primary objective of this consensus statement is to improve diagnosis and treatment of allergic rhinitis in primary care, and offer guidance on appropriate referral of difficult-to-treat patients into secondary care. The guidance provided herein outlines a sequential treatment pathway for allergic rhinitis in primary care that incorporates a considered approach to improve the management of allergic rhinitis symptoms and improve compliance and patient satisfaction with therapy. Adherence with this care pathway has the potential to limit the cost of providing effective allergic rhinitis management in the UK by avoiding unnecessary treatments and investigations, and avoiding the need for costly referrals to secondary care in the majority of allergic rhinitis cases. The fundamentals presented in this consensus article should apply in most health-care settings.

## Introduction

The aim of any disease management plan is control of the disease. In 2015, a multi-professional group of clinicians met in Stirling, Scotland to discuss the development of a simplified allergic rhinitis (AR) treatment algorithm for use in primary and secondary care, incorporating current knowledge and recently available treatments. This consensus statement is a result of that initial meeting and subsequent collaboration among the working party. The primary objective is to promote better management of AR patients, improve diagnosis and treatment in primary care and to facilitate transition of difficult-to-treat patients into secondary care.

## How AR can affect patients?

AR a common condition that affects >20% of the UK population, is a frequent presenting problem in primary care,^1,2^ and is increasing in prevalence.^3,4^ It is associated with considerable morbidity; significantly reducing quality of life,^5,6^ interfering with attendance and performance at school and work,^7–9^ and resulting in considerable health-care and indirect costs.^10–13^ Around 50–80% of AR patients report that their condition interferes with sleep, resulting in daytime fatigue, decreased alertness, inability to concentrate, depression and irritability.^14^ AR is also associated with a number of coexisting conditions, including asthma, sinusitis, upper respiratory tract infection, otitis media with effusion and nasal polyposis.^15,16^

## AR management in the UK

Management of AR is generally not allergen-specific and relies in the main on pharmacotherapy to control symptoms.^17,18^ Most mild-moderate AR symptoms can be managed successfully in primary care. However, one-third of general practitioner (GP) specialty training programmes do not provide allergy training.^19^ Initial AR monotherapy is failing many patients in the UK.^20^ A database survey of 22,000 AR patients living in the UK showed that the majority of seasonal AR (SAR) and perennial AR (PAR) patients received monotherapy as their initial prescription of the season, but such monotherapy (e.g., antihistamine [AH] or intranasal corticosteroid [INCS]) proved insufficient for many (20–25% SAR patients; 37–46% of PAR patients). Other patients started the season on a multi-therapy regimen (33% and 23% of SAR and PAR patients, respectively) and this proportion increased during the season. As the number of therapies prescribed increased, so too did the number of return GP consultations. By the end of the season around half of all AR sufferers were prescribed multiple therapies, most commonly oral AH plus INCS. However, this combination is not supported by clinical trials of combination regimens vs. monotherapy.^21,22^ The Allergic Rhinitis and its Impact on Asthma (ARIA) guidelines state that “combination between drugs has been tested, but insufficient data are available to make a recommendation concerning the combined use of oral AH and INCS”.^23^

## Why it’s important to get rhinitis under control

The link between AR and asthma is well-established.^23,24^ Both AR and non-AR are risk factors for the development of asthma; co-morbid rhinitis affects up to 75% of those with asthma.^24,25^ Poor control of rhinitis predicts poor control of asthma; asthma patients with significant rhinitis are four-times more likely to have poorly-controlled asthma than those without,^24^ with a negative impact on asthma control equivalent to that of smoking.^26^ Moreover, failure to treat AR appropriately results in increased asthma medication use for co-morbid patients.^27^

## Assessment of AR control

Some sophisticated AR control questionnaires have been developed including the Control of Allergic Rhinitis and Asthma Test^28^ and Rhinitis Control Assessment Test.^29,30^ Others advocate a multi-factorial assessment incorporating an assessment of (i) severity and/or frequency of daily or nocturnal symptoms, (ii) impairments in social, physical, professional and educational activities, (iii) respiratory function monitoring and (iv) exacerbations (e.g., unscheduled medical consultations and rescue medication use).^31^ Although useful in clinical trials these are unlikely to be used routinely in day-to-day practice. ARIA has recently recommended a simple visual analogue scale (VAS) as the new language of AR control, with a score of 5/10 cm used to assess control and guide treatment decisions as part of a simple algorithm called the AR clinical decisions support system (CDSS).^32^ This VAS has also been incorporated into an app for patients (*Allery Diary*). A companion app for physicians is currently under development (which includes the AR CDSS; *Allergy Diary Companion*), thus linking key stakeholders with a common language of AR control.

## What AR patients want from treatment

Living with symptomatic AR means coping with any or all of the symptoms of nasal congestion, headache, postnasal drip, repeated sneezing, runny nose and other symptoms on a near-daily basis.^33^ AR patients have high expectations from their treatment,^34^ but also a high degree of dissatisfaction with AR treatment,^35^ for reasons that include: symptom breakthrough, lack of 24-hour coverage, lack of coverage of both nasal and ocular symptoms, and unwanted side effects.^15^ Patients want faster and more complete symptom control.^27^ Insufficient symptom control with INCS and oral AH is a major concern, and a situation, which has not improved over time.^15^ Many physicians underestimate AR severity and consequently fail to issue adequate treatment,^36^ a situation which appears to have changed little in a decade.^37^

## Management in primary care—diagnosis

**Fig. 1:**
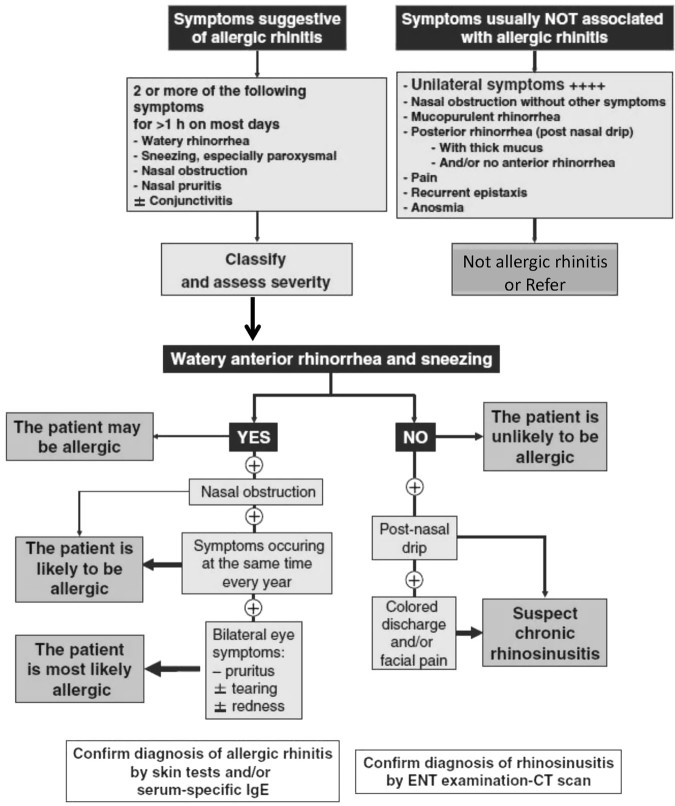
Diagnosis of allergic rhinitis through symptom assessment. Adapted from ARIA Bousquet 2008.^23^ IgE: immunoglobulin E; ENT: Ear Nose Throat; CT: computed tomography

The definition, aetiology and classifications of AR are described in Table 1,^1,23^ and the diagnosis of AR by symptom assessment is outlined in Fig. 1.^23^ Confirmation of the diagnosis may be made through questions concerning family history, social history (housing, pets, occupation, possible triggers), and visual examination. The common triggers for AR are listed in Table 1.^1^

**Table 1:** Definition, aetiology, classification and common triggers of allergic rhinitis (AR)

**AR—definition**	**Defining symptoms**	**Characteristics**
A symptomatic disorder of the nose induced after allergen exposure by an IgE-mediated inflammation	Rhinorrhoea, nasal obstruction, nasal itching and sneezing	Symptoms are reversible spontaneously or with treatment
**Aetiology**	**Immediate reaction**	**Late-phase reaction**
TH2-mediated inflammation	Rapid IgE-mediated mast cell degranulation and mediator release	Inflammation, with an eosinophilic infiltrate
**Classifications of AR**		
*Seasonal*: in response to a seasonal allergen (e.g., tree pollen)	*Perennial*: in response to an allergen present all year round (e.g., HDM)	
*Intermittent*: Symptoms are present <4 days/week OR for <4 consecutive weeks	*Persistent*: Symptoms are present >4 days/week AND for >4 consecutive weeks	
**Severity of AR**		
*Mild*– No factors are present	*Moderate/Severe*– One or more factors are present	*Factors:* • Sleep disturbance • Impairment of daily activities, leisure and/or sport • Impairment of school/work • Troublesome symptoms
**Common triggers**		
*Trigger type*	*Examples of triggers*	*Types of AR*
Mites	HDM, storage mites	Perennial
Pollens	Trees, grasses, shrubs, weeds	Seasonal
Animals	Cats, dogs, horses, rodents	Perennial
Moulds	*Alternaria, Cladosporium, Aspergillus*	Seasonal and/or Perennial
Occupational	Flour, latex, laboratory animals, wood dust, chlorine, chloramine, enzymes, other airborne proteins	Reversible with early diagnosis and avoidance but becomes chronic and irreversible

Adapted from Scadding 2008; Bousquet 2008^1,23^

*IgE* Immunoglobulin E, *TH2* T-helper-2, *HDM* house dust mite

AR is the predominant form of rhinitis in children, but also accounts for one-third of adult cases.^1^ At greatest risk are those with a personal or family history of atopy (defined by positive skin prick tests or specific IgE to common aeroallergens).^2^ The clinical history should determine whether allergy testing is required, which may be useful to identify or exclude an allergic trigger that may influence management, e.g., aeroallergen avoidance. GPs should perform either skin prick testing or serum total/specific IgE depending on local availability. The former has the advantage that it is cheaper and the patient gets instant feedback, but is time consuming and requires stopping anti-histamines for a week. Ideally, allergy testing would be performed in all patients with rhinitis not only for advice about potential allergen avoidance but also because it will alter the management pathway. Allergy testing will not, however, identify non antigenic chemical irritants which can cause nasal airway hyper-reactivity through a non IgE-mediated pathway. Indeed in many cases exogenous chemical irritants may aggravate underlying AR. In reality, the majority of patients presenting in primary care with AR can be managed without formal identification of the specific allergic trigger^18^ with pharmacotherapy.

## Management in primary care—treatment

**Fig. 2:**
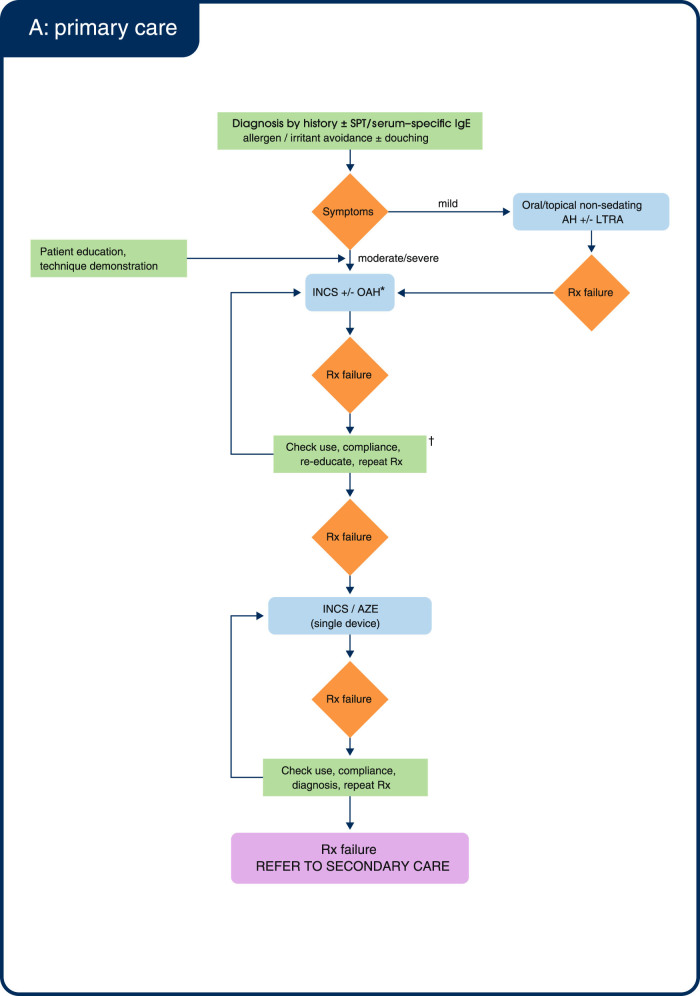

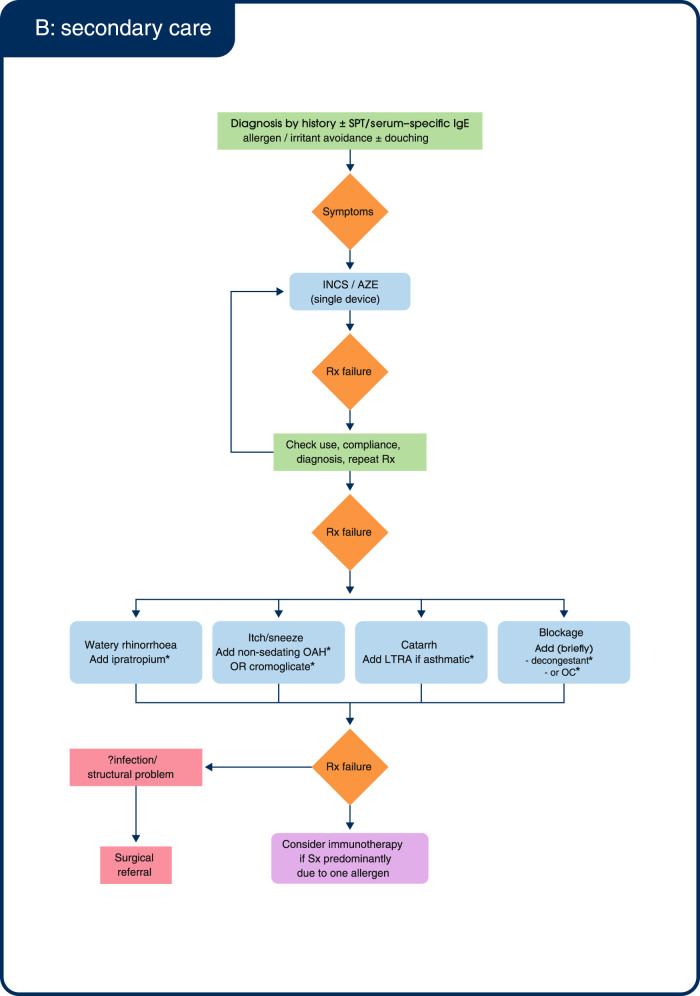
Treatment algorithm for allergic rhinitis in (**a**) primary care and (**b**) secondary care. AH: anti-histamine; AZE: azelastine; IgE: immunoglobulin E; INCS: intranasal corticosteroid; LTRA: leukotriene receptor antagonist; OAH: oral antihistamine; OC: oral corticosteroid; Rx: treatment; SPT: skin prick test; Sx: symptoms. **(a)** *You may consider the addition of an OAH to INCS. However, scientific evidence shows that adding an OAH to INCS provides no additional benefit over INCS alone.^21, 45^^†^This consensus group does not recommend routine early use of oral corticosteroids (OC); OC use should be reserved for short-term acute severe symptoms. **(b)** *Evidence for the benefit of these add-ons is lacking. Adapted from BSACI Scadding 2008.^1^

Figure 2a shows an algorithm for the treatment of AR in primary care, adapted by the consensus group from the 2008 British Society of Allergy & Clinical Immunology (BSACI) guidelines^1^ in order to simplify treatment in primary care and incorporate recently available treatment options.

### Education

Patients should be educated on the nature of AR, allergen avoidance, available treatments (including safety and potential side effects), and be given realistic expectations of the results of therapy. Treatment failure may be related to poor technique in the use of nasal sprays and drops and, therefore, appropriate training is essential.

### Treatment options

#### Nasal douching

Saline douching is a safe, inexpensive treatment, demonstrated to reduce symptoms in children and adults with seasonal rhinitis.^38,39^ It is more commonly used in mainland Europe than in the UK. In our opinion it has only a limited role in the management of AR, and there is little objective data to support its use.

#### Oral/topical non-sedating antihistamines

Oral and topical non-sedating AHs are recommended as first-line therapy for mild-moderate intermittent and mild persistent AR. The therapeutic effect of intranasal AH is superior to that that of oral AH,^40^ with the additional benefit of a faster onset of action (i.e., within 15 mins).^41^ Patients responsive to AH therapy should be instructed to continue therapy during periods of allergen exposure. Those non-responsive to therapy (e.g., symptomatic after 2 weeks) should be stepped up.

#### Intranasal corticosteroids

INCSs are recommended as first-line therapy for moderate-severe persistent symptoms and treatment failures with AHs alone. For patients who do not tolerate INCSs or prefer not to use them, adding a leukotriene receptor antagonist to an oral or topical AH may be considered.^42,43^ Responsive patients should be instructed to continue therapy during periods of allergen exposure. Those non-responsive to therapy (e.g., symptomatic after 4 weeks) should be stepped up.

#### Recently-approved AR treatment

The combination of intranasal azelastine hydrochloride (AZE) and fluticasone propionate (FP) in a single device (Dymista® (INCS/AZE); Meda, Solna, Sweden) is a novel formulation that is indicated for adults and adolescents ≥12 years with moderate/severe SAR or PAR if monotherapy with either INCS or AH is not considered sufficient.^44^ In primary care, the consensus group considers that INCS/AZE (single device) may be used second line after failure of topical INCS after checking for the correct use and compliance with medication. If incorrect use or non-compliance is found, re-education followed by repeat trial of topical INCS is recommended before INCS/AZE (single device) prescription. Alternatively, the addition of an oral AH to INCS may be considered in the case of INCS monotherapy failure, particularly for children <12 years for whom INCS/AZE (single device) is not yet indicated. However, it should be noted that the evidence to support this practice is lacking.^21,45^

INCS/AZE (single device) may be used first line in certain cases in which the GP considers that monotherapy is more likely to fail, if they consider it warranted in their professional opinion, in accordance with the labelled product indication.^44^ This recommendation is based on an extensive review of existing clinical trial evidence,^46–49^ and data obtained in routine clinical practice in several countries in Europe including Germany, Sweden, Denmark, Norway and Romania.^50^ INCS/AZE (single device) is recommended first-line in the recently published AR CDSS from ARIA for those patients with a VAS score >5/10 cm.^32^ The speed of onset with INCS/AZE (single device) for both nasal and ocular symptom relief and the use of a single spray may improve compliance and treatment outcomes by avoiding administration and delivery issues.^51^

#### Oral corticosteroids

This consensus group does not recommend routine early use of oral corticosteroids (OC), which should be reserved for short-term acute severe symptoms, in combination with a topical INCS or AZE/INCS (single device). A suggested regime for adults is 0.5 mg/kg given orally in the morning for 5–10 days.^1^

### Referral

In the event of treatment failure on second-line INCS/AZE (single device) in primary care, checking for the correct use of medication, compliance, and whether the correct diagnosis was made is advised. Following that, referral to secondary care is recommended for further investigations.

### Management in secondary care

In secondary care, the consensus group considers that INCS/AZE (single device) could be used as a first-line alternative to topical INCS in patients with moderate-severe symptoms (Fig. 2b). This is in contrast to primary care where high bioavailability INCS (e.g., beclomethasone dipropionate) first line is often prescribed based on formulary recommendation and acquisition cost rather than on efficacy, safety or patient-reported outcomes. The consensus group, however, had a preference for modern, low bioavailability steroids (e.g., budesonide; FP, fluticasone furoate or mometasone furoate).

Symptom-specific investigations for refractory symptoms should be completed in secondary care following INCS/AZE (single device). Add-on therapy with symptom-specific treatment such as ipratropium for watery rhinorrhoea, or oral AH for nasal itching/sneezing may be reserved for use in secondary care. It should be noted that currently there is no scientific evidence to support this practice.^21,45^ In the event of INCS/AZE (single device) failure, the consensus group recommended adding OC for 7–14 days for control of acute severe symptoms, for example during the peak of the pollen season. If treatment failure continues, the diagnosis should be revisited in the first instance, while surgery or immunotherapy may be subsequent treatment options.

It seems likely that the use of INCS/AZE (single device) may be an additional treatment option prior to immunotherapy. Immunotherapy may be appropriate for some patients with persistent symptoms predominantly due to one allergen such as grass pollen. However, many patients are polysensitised and will remain symptomatic despite immunotherapy; these patients should receive symptomatic treatment in addition to immunotherapy. Relapse following successful immunotherapy treatment may occur; for these patients further treatment options including symptomatic treatment may be considered.

## Discussion

Achieving adequate symptom control is pivotal to successful AR management and may be attained though the stepwise treatment algorithm outlined here. Correct administration technique for INCS and good compliance with treatment regimen are essential to achieve symptom control, and should be checked in the event of early treatment failures. In primary care, following treatment failure on INCS the consensus panel does not recommend increasing the dose of INCS, for which there is little evidence due to the recognised ceiling effect.^47^ In addition, switching INCS is also not recommended as all have comparable efficacy.^52–54^ The panel recommends prescription of INCS/ AZE (single device) as the evidence-based option,^46–49^ rather than adding oral AH to INCS, as there is lack of consistent evidence for the additive efficacy of the latter.^21,45^ The consensus panel judge that positioning INCS/AZE (single device) as a second-line intervention in the primary care pathway may provide value to the health service by averting repeat GP visits (at £45 per consultation)^27^ or costly secondary care referrals (at approximate costs of £136 for ENT referral, and £212 for paediatric referral).^55^ The fundamentals presented in this consensus article should apply in most health-care settings, although obviously different patterns of allergen exposure and allergens will occur, and cost considerations and pharmacotherapy availability may differ.

Achieving adequate symptom control through efficacious prescription medication will avoid patients resorting to self-medication with add-on over-the-counter (OTC) products, which can lead to adverse effects with prolonged use, e.g., rhinitis medicamentosa with repeat topical nasal decongestants such as oxymetazoline.^56^ There are patients who do not tolerate the first-generation oral AHs available OTC (e.g., chlorpheniramine), and the safety of drivers who self-medicate on escalating doses of additive first-generation oral AHs is a concern. In addition, there is a need for alternatives to OCs for symptom relief for major life events, such as exams and weddings. The panel considered that OCs are positioned too early in the 2008 BSACI algorithm,^1^ and should be recommended only for short-term use to gain control of acute severe symptoms. The forthcoming update to the BSACI guidelines is awaited with interest.

It was noted that all current guidelines take a patient down a route of escalating treatment without taking a step back and assessing whether treatment can be stepped down and when the patient should be referred. Up to 50% of AR patients in primary care are receiving step 3 treatment with polypharmacy, perhaps inappropriately.^27^ A proportion of patients will not be adequately controlled on initial therapies, and should be referred to secondary care for confirmation of diagnosis and further investigation. They may also receive symptom-specific add-on therapy. There is some advocacy for a step down approach to AR management; for example, starting with INCS/AZE (single device) in patients with previous treatment failure or resistance to monotherapy. After a few weeks of achieving complete control, consideration could be given to treatment reduction e.g., stepping down to INCS alone. However, it is acknowledged that the step-down approach is based on consensus and more data are needed. Nonetheless, positioning INCS/AZE (single device) first line in secondary care with an integrated check of compliance and technique has the potential to avert costly add-on treatments, repeat visits and possibly the need for immunotherapy or surgery.

## Conclusion

With the availability of effective therapy, the majority of AR symptoms can be treated in the primary care setting. This guidance provides a clear pathway for AR treatment in primary care practice that outlines a considered approach to improve the management and control of AR symptoms and improve compliance and patient satisfaction with therapy. Adherence with this care pathway has the potential to limit the cost of providing effective AR management in the UK by avoiding unnecessary treatments, and investigations and avoiding the need for unnecessary repeat GP visits and costly referrals to secondary care in the majority of AR cases.

## References

[1] Scadding GK (2008). BSACI guidelines for the management of allergic and non-allergic rhinitis. Clin. Exp. Allergy.

[2] Angier E, Willington J, Scadding G, Holmes S, Walker S (2010). Management of allergic and non-allergic rhinitis: a primary care summary of the BSACI guideline. Prim. Care Respir. J..

[3] Aberg N, Hesselmar B, Aberg B, Eriksson B (1995). Increase of asthma, allergic rhinitis and eczema in Swedish schoolchildren between 1979 and 1991. Clin. Exp. Allergy.

[4] Maziak W (2003). Are asthma and allergies in children and adolescents increasing? Results from ISAAC phase I and phase III surveys in Munster, Germany. Allergy.

[5] Bousquet PJ, Demoly P, Devillier P, Mesbah K, Bousquet. J (2013). Impact of allergic rhinitis symptoms on quality of life in primary care. Int. Arch. Allergy Immunol..

[6] Small M, Piercy J, Demoly P, Marsden H (2013). Burden of illness and quality of life in patients being treated for seasonal allergic rhinitis: a cohort survey. Clin. Transl. Allergy.

[7] Walker S (2007). Seasonal allergic rhinitis is associated with a detrimental effect on examination performance in United Kingdom teenagers: case-control study. J. Allergy Clin. Immunol..

[8] Blaiss MS (2004). Allergic rhinitis and impairment issues in schoolchildren: a consensus report. Curr. Med. Res. Opin..

[9] Szeinbach SL, Seoane-Vazquez EC, Beyer A, Williams PB (2007). The impact of allergic rhinitis on work productivity. Prim. Care Respir. J..

[10] Blaiss MS (2000). Cognitive, social, and economic costs of allergic rhinitis. Allergy. Asthma. Proc..

[11] Lamb CE (2006). Economic impact of workplace productivity losses due to allergic rhinitis compared with select medical conditions in the United States from an employer perspective. Curr. Med. Res. Opin..

[12] Hellgren J, Cervin A, Nordling S, Bergman A, Cardell LO (2010). Allergic rhinitis and the common cold–high cost to society. Allergy..

[13] de la Hoz CB (2012). Allergic rhinitis and its impact on work productivity in primary care practice and a comparison with other common diseases: the Cross-sectional study to evAluate work Productivity in allergic Rhinitis compared with other common diseases (CAPRI) study. Am. J. Rhinol. Allergy.

[14] Storms W (2008). Allergic rhinitis-induced nasal congestion: its impact on sleep quality. Prim. Care Respir. J..

[15] Nathan RA (2007). The burden of allergic rhinitis. Allergy Asthma. Proc..

[16] Schatz M (2008). The burden of rhinitis in a managed care organization. Ann. Allergy Asthma Immunol..

[17] Ryan D, Levy M, Morris A, Sheikh A, Walker S (2005). Management of allergic problems in primary care: time for a rethink?. Prim. Care Respir. J..

[18] Walker S, Morton C, Sheikh A (2006). Diagnosing allergy in primary care: are the history and clinical examination sufficient?. Prim. Care Respir. J..

[19] Ellis J, Rafi I, Smith H, Sheikh A (2013). Identifying current training provision and future training needs in allergy available for UK general practice trainees: national cross-sectional survey of General Practitioner Specialist Training programme directors. Prim. Care Respir. J..

[20] Price D (2016). UK prescribing practices as proxy markers of unmet need in allergic rhinitis: a retrospective observational study. NPJ Prim. Care Respir. Med..

[21] Anolik R, Mometasone Furoate Nasal Spray With Loratadine Study Group (2008). Clinical benefits of combination treatment with mometasone furoate nasal spray and loratadine vs monotherapy with mometasone furoate in the treatment of seasonal allergic rhinitis. Ann. Allergy Asthma Immunol..

[22] Esteitie R, deTineo M, Naclerio RM, Baroody FM (2010). Effect of the addition of montelukast to fluticasone propionate for the treatment of perennial allergic rhinitis. Ann. Allergy Asthma Immunol..

[23] Bousquet J (2008). Allergic Rhinitis and its Impact on Asthma (ARIA) 2008 update (in collaboration with the World Health Organization, GA(2)LEN and AllerGen). Allergy..

[24] Scadding G, Walker S (2012). Poor asthma control?–then look up the nose. The importance of co-morbid rhinitis in patients with asthma. Prim. Care Respir. J..

[25] Bachert C (2004). Allergic rhinitis, rhinosinusitis, and asthma: one airway disease. Immunol. Allergy Clin. North. Am..

[26] Clatworthy J, Price D, Ryan D, Haughney J, Horne R (2009). The value of self-report assessment of adherence, rhinitis and smoking in relation to asthma control. Prim. Care Respir. J..

[27] Price D (2015). The hidden burden of adult allergic rhinitis: UK healthcare resource utilisation survey. Clin. Exp. Allergy.

[28] Azevedo P (2013). Control of allergic rhinitis and asthma test (CARAT): dissemination and application in primary care. Prim. Care Respir. J..

[29] Nathan RA (2014). The rhinitis control assessment test: implications for the present and future. Curr. Opin. Allergy Clin. Immunol..

[30] Nathan RA (2010). Qualitative development of the Rhinitis Control Assessment Test (RCAT), an instrument for evaluating rhinitis symptom control. Patient.

[31] Demoly P (2013). Assessment of disease control in allergic rhinitis. Clin. Transl. Allergy.

[32] Bousquet J (2016). MACVIA clinical decision algorithm in adolescents and adults with allergic rhinitis. J. Allergy. Clin. Immunol..

[33] Meltzer EO (2009). Burden of allergic rhinitis: results from the Pediatric Allergies in America survey. J. Allergy Clin. Immunol..

[34] Hellings PW (2012). Explorative study on patient's perceived knowledge level, expectations, preferences and fear of side effects for treatment for allergic rhinitis. Clin. Transl. Allergy.

[35] Ciprandi G (2011). Patient-related factors in rhinitis and asthma: the satisfaction with allergy treatment survey. Curr. Med. Res. Opin..

[36] Gronhoj LC, Gyldenlove M, Linneberg A (2013). Allergic rhinitis is often undiagnosed and untreated: results from a general population study of Danish adults. Clin. Respir. J..

[37] Ryan D (2005). Management of allergic rhinitis in UK primary care: baseline audit. Prim. Care. Respir. J..

[38] Garavello W, Di BF, Romagnoli M, Sambataro G, Gaini RM (2005). Nasal rinsing with hypertonic solution: an adjunctive treatment for pediatric seasonal allergic rhinoconjunctivitis. Int. Arch. Allergy Immunol..

[39] Tomooka LT, Murphy C, Davidson TM (2000). Clinical study and literature review of nasal irrigation. Laryngoscope..

[40] Horak F, Zieglmayer UP (2009). Azelastine nasal spray for the treatment of allergic and nonallergic rhinitis. Expert. Rev. Clin. Immunol..

[41] Ellis AK, Zhu Y, Steacy LM, Walker T, Day JH (2013). A four-way, double-blind, randomized, placebo controlled study to determine the efficacy and speed of azelastine nasal spray, versus loratadine, and cetirizine in adult subjects with allergen-induced seasonal allergic rhinitis. Allergy. Asthma. Clin. Immunol..

[42] Erdogan BA, Sanli A, Paksoy M, Altin G, Aydin S (2014). Quality of life in patients with persistent allergic rhinitis treated with desloratadine monotherapy or desloratadine plus montelucast combination. Kulak. Burun. Bogaz. Ihtis. Derg..

[43] Cingi C (2013). Desloratadine-montelukast combination improves quality of life and decreases nasal obstruction in patients with perennial allergic rhinitis. Int. Forum. Allergy Rhinol..

[44] Dymista summary of product characteristics. https://www medicines org uk/emc/medicine/27579 2014. [last accessed 02.09.16]

[45] Di Lorenzo G (2004). Randomized placebo-controlled trial comparing fluticasone aqueous nasal spray in mono-therapy, fluticasone plus cetirizine, fluticasone plus montelukast and cetirizine plus montelukast for seasonal allergic rhinitis. Clin. Exp. Allergy.

[46] Carr W (2012). A novel intranasal therapy of azelastine with fluticasone for the treatment of allergic rhinitis. J. Allergy Clin. Immunol..

[47] Meltzer E (2013). Clinically relevant effect of a new intranasal therapy (MP29-02) in allergic rhinitis assessed by responder analysis. Int. Arch. Allergy Immunol..

[48] Price D (2013). A new therapy (MP29-02) is effective for the long-term treatment of chronic rhinitis. J. Investig. Allergol. Clin. Immunol..

[49] Berger WE (2014). Long-term, randomized safety study of MP29-02 (a novel intranasal formulation of azelastine hydrochloride and fluticasone propionate in an advanced delivery system) in subjects with chronic rhinitis. J. Allergy Clin. Immunol. Pract..

[50] Klimek, L. *et al.* MP-AzeFlu provides rapid and effective allergic rhinitis control in real-life: a pan-European study. *Allergy Asthma Proc.* **37**, 376–386 (2016). 10.2500/aap.2016.37.3979 27657521

[51] D'Addio A (2014). Deposition characterisitcs of a new allergic rhinitis nasal spray (MP29-02) in an anatomical model of the human nasal cavity. Allergy..

[52] Aneeza WH (2013). Efficacy of mometasone furoate and fluticasone furoate on persistent allergic rhinoconjunctivitis. Allergy Rhinol (Providence).

[53] Okubo K, Nakashima M, Miyake N, Komatsubara M, Okuda M (2009). Comparison of fluticasone furoate and fluticasone propionate for the treatment of Japanese cedar pollinosis. Allergy Asthma Proc..

[54] Mandl M, Nolop K, Lutsky BN (1997). Comparison of once daily mometasone furoate (Nasonex) and fluticasone propionate aqueous nasal sprays for the treatment of perennial rhinitis. 194-079 Study Group. Ann. Allergy Asthma Immunol..

[55] Unit costs of health and social care. https://www.gov.uk/government/publications/national-tariff-payment-system-2014-to-2015. [last accessed 10.11.15]

[56] Graf P (2005). Rhinitis medicamentosa: a review of causes and treatment. Treat. Respir. Med..

